# Training pediatric residents on navigating moral events in contemporary ethical practice: curriculum design and evaluation

**DOI:** 10.1186/s12910-026-01555-8

**Published:** 2026-07-17

**Authors:** Chinyere O’Connor, Hunter Hamilton, Amanda McCarthy, Margarita Ortiz, S. Shahrukh Hashmi, Sienna Wu, Shriya Sachdeva, Kaleigh Riggs- Harpur, Aymen Mirza, Michelle S. Barratt, Emma Omoruyi, Terri Major-Kincade

**Affiliations:** 1 Department of Pediatrics, Division of Pediatric Critical Care, McGovern Medical School at UTHealth Houston, 1133 John Freeman Blvd, Suite JJL 205 N, Houston, TX 77030 USA; 2 Palliative Care Division, Division of Pediatric Critical Care, Department of Pediatrics, McGovern Medical School at UTHealth Houston, 6431 Fannin St, Houston, TX 77030 USA; 3 McGovern Medical School at UTHealth Houston, McGovern Center for Humanities and Ethics, 6431 Fannin St, Houston, TX 77030 USA; 4 McGovern Medical School at UTHealth Houston, 6431 Fannin St, Houston, TX 77030 USA; 5 Department of Pediatrics, Division of General Academic Pediatrics, McGovern Medical School at UTHealth Houston, 6431 Fannin St, Houston, TX 77030 USA; 6 Department of Pediatrics, Pediatric Palliative Medicine, Division of Neonatal-Perinatal Medicine, McGovern Medical School at UTHealth Houston, 6431 Fannin St, Houston, TX 77030 USA

**Keywords:** Moral events, Moral distress, Ethical dilemmas, Professionalism, Curriculum, Curriculum evaluation, Simulated encounters

## Abstract

**Background:**

Morally challenging situations arise frequently in contemporary pediatric practice. Despite this, few pediatric studies have examined how to support residents navigating these experiences or measure growth in this domain. To address this gap, we developed and piloted a longitudinal, multimodal curriculum designed to prepare residents to recognize and respond to moral challenges.

**Methods:**

Curriculum development followed Kern's six-step model and was informed by a previously published needs assessment and review of existing literature. A mixed-methods design evaluated the intervention across Kirkpatrick's levels of evaluation. We designed two complementary tools: (a) an Objective Structured Clinical Examination (OSCE) emphasizing applied ethical reasoning and professional accountability, and (b) the Cognitive Affective Reflection on Ethical Experiences: Moral Encounters Tool (CARE-MET), which provided preliminary validity evidence for assessing residents' perceived self-efficacy in navigating moral events. Thematic and content analyses were conducted on qualitative data. Paired analyses of matched participants at timepoint 1 (T1) and timepoint 2 (T2) were conducted using Wilcoxon Rank Sum test and Cohen's *d* effect sizes.

**Results:**

Of 81 eligible residents, 65 completed the CARE-MET at T1 and 52 of 57 eligible residents completed it at T2. Most participants (64/65, 97%) rated the curriculum as valuable. Residents identified value in the use of real clinical cases, diverse perspectives, and protected space for emotional processing. Paired analyses revealed significant improvements across five of six CARE-MET domains with large Cohens *d* (> 0.7). Experiencing moral distress was unchanged pre- and post-curriculum. Two separate cohorts of PGY-3 residents completed the OSCE at T1 (*n* = 12) and T2 (*n* = 14). Residents in both cohorts scored similarly high in empathic practice. Communicating the possibility of death without treatment was one significant difference between cohorts. This shift occurred more frequently at T2 than T1 (36% vs 0%, *p* = 0.024).

**Conclusions:**

This curriculum offers a promising framework for preparing pediatric residents to navigate care that might be morally distressing yet ethically permissible. Gaps persisted in applied decision-making, legal understanding, and professional accountability. These findings support the need for longitudinal instruction in the moral, ethical, and legal dimensions of pediatric practice and highlight the value of developing tools capable of measuring growth in these domains.

## Introduction

The Accreditation Council of Graduate Medical Education (ACGME) requires that training programs enhance "residents' knowledge of ethical principles foundational to medical professionalism” [1]. Formal ethics curricula exist to address this mandate, but pediatric practice poses distinct challenges [2–4]. Residents engage with a vulnerable population (minors) and their legal guardians, each holding differing perspectives on what is “good,” “right,” or “desirable” [5]. Professional, ethical, and legal guidance frequently diverge, especially under the pressures of contemporary practice. Moral obligations, an added fourth dimension in care, have become more salient in a pluralistic society where beliefs surrounding best interests, beneficence, and non-maleficence vary among caregivers. Tensions from these competing obligations are further amplified for residents by their limited agency and frequent positioning as bystanders to the moral event.

Moral events are frequently conflated with ethical dilemmas. Ethical dilemmas are clinical situations in which competing ethical, professional, or legal obligations create uncertainty about the “right” course of action [6–8]. Moral events, on the other hand, encompass experiences of moral tension, conflict, uncertainty, dilemma, or constraint. They may arise even when an ethically permissible course of action exists and often produce moral distress, defined as the psychological discomfort that arises as a result of the moral event [9, 10]. If not addressed, moral distress can accumulate into moral residue and contribute to burnout [9, 10].

Decades ago, evidence linked inadequate ethics training to low confidence in end-of-life (EOL) decision-making [11, 12]. While EOL care represents a profound moral event for pediatric trainees, far less attention has been given to navigating the moral events that arise from the accelerated pace of medical and technological advancement [5, 13, 14]. Teaching through complex moral events, which may or may not involve an ethical dilemma, can also provide a concrete pathway for reinforcing professionalism [15]. Practicing within the ethical dimensions of pediatric care requires residents to integrate communication skills, moral and ethical reasoning, legal awareness, and accountability [16]. These same competencies underpin core professionalism expectations, and unsupported moral events may erode residents’ capacity for empathy and sensitivity. The limited impact of existing ethics and humanities curricula may in part reflect insufficient attention to moral distress [5]. Consequently, teaching residents to reason through ethical dimensions of care and reflect on their emotional responses offers a unified approach to advancing both ethical and professionalism development.

To ensure that such training achieves its intended outcomes, systematic evaluation warrants the same rigor as curriculum design. Traditional assessments of ethics and professionalism have relied largely on self‑evaluations and the “hidden curriculum,” which provide limited validity evidence and rarely translate into observable outcomes [13, 17–21]. Other fields have implemented innovative assessment modalities through high‑fidelity simulation, standardized patients, bereaved parents, and longitudinal mentorship curricula [22–30]. In pediatrics, however, few studies have examined innovative approaches such as Objective Structured Clinical Examinations (OSCEs) to systematically teach and assess the interconnectedness of morality, the ethical dimensions of care, and professionalism [31].

To address the gaps in preparing residents to recognize and respond to ethics related moral events, we had two specific aims: (1) Implement Kern’s 6-step approach to design and create a *needs driven* curriculum focused on navigating moral events in pediatric practice, and (2) Demonstrate the effectiveness of the curriculum using all four Kirkpatrick's levels of evaluation [32, 33].

## Methods

### Design

This was a prospective mixed-methods study evaluating the implementation and impact of a pediatric curriculum focused on navigating ethics related moral events. Curriculum development followed Kern’s six-step model, and evaluation was guided by Kirkpatrick’s four levels of evaluation outcomes [32, 33].

### Participants

Categorical pediatric residents at UTHealth Houston were eligible to participate in the curriculum. Residents across all PGY levels were invited to complete pre- and post-curriculum surveys. Two separate cohorts of PGY-3 residents completed the OSCE during protected educational time, one prior to curriculum implementation and one following it. Approval for this study was obtained from the Institutional Review Board (HSC-MS-22–0891). The curriculum became a required component of residents’ education while assessment instruments remained voluntary. All participants who completed assessment instruments provided informed consent.

### Curriculum development (Kern’s six steps)

#### Problem and needs assessment (step 1 and 2)

A 2020 retrospective chart review showed that while fewer than 1% of PGY-1 residents encountered a patient death, 96% of PGY-2 and 78% of PGY-3 residents did. These encounters were most often in intensive care and oncology settings [34]. With “curricular crowding” and time constraints common barriers to educational interventions, and even less time available for ethics related interventions, needs assessments can be useful for setting educational priorities. Thus, to better understand the ethical and emotional challenges residents face in these environments, an initial needs assessment consisting of monthly handwritten reflective exercises was conducted from January to June 2023 with rotating groups of pediatric residents in the Pediatric Intensive Care Unit (PICU). The handwritten format encouraged tactile engagement and authenticity, capturing in-the-moment responses. An interpretive and descriptive qualitative approach analyzed the data. Detailed results from our needs assessment are published elsewhere [35].

#### Goals and objectives (step 3)

Moral dimensions that emerged from the needs assessment included: balancing clinical care with quality of life, emotional burden communicating challenging news to families, tension between parental expectations and clinical realities, managing personal emotions with professional boundaries, and poor collaboration with other clinical teams. Residents described ethical conflict using terms such as ‘uncomfortable,’ ‘distress,’ ‘powerless,’ ‘frustrating,’ ‘learning opportunity’ ‘emotionally exhausting,’ and ‘uncertainty’ [35]. Our findings informed the development of targeted goals and objectives which aligned with Entrustable Professional Activity 2 and ACGME competencies in patient- and family-centered communication, but extended into the moral and ethical dimensions of care [36, 37]. The curriculum emphasized moral reasoning, values negotiation, emotional self-awareness and resiliency as core components of professional practice. Specifically, our learner-centered goals were: (a) ensure all graduating residents possess skills on exploring, navigating and distinguishing between moral events and real or perceived ethical dilemmas regardless of their future career path; (b) guide trainees in recognizing and critically analyzing ethical principles in medical practice that affect patient care and provide educational infrastructure that allows residents to voice and share in values clarification with their peers; and (c) emphasize empathy as a core dimension of care by identifying and examining personal assumptions, exploring value conflicts, addressing moral distress, and tolerance of opposing viewpoints.

#### Educational strategies and implementation (step 4 and 5)

The curriculum content was informed by both our needs assessment findings and a review of existing literature on ethics education, moral distress, and professionalism development in pediatric residency training [13–15, 34, 35, 38]. The curriculum was delivered over 12 months (August 2023 to August 2024) through two formats: a large group session series and a small group session series with at least one session offered monthly. Each session was self-contained, did not require prereading, and was incorporated into existing protected educational time. These didactic sessions implemented semi-structured modules built around familiar clinical cases designed to surface ethical tensions and raw emotional responses. Sessions followed adult learning theory moving from pedagogy to andragogy emphasizing self-reflection, relevance of content to learners, and addressing intrinsic motivators to learn [39]. We delivered the curriculum through a multimodal format that included interdisciplinary facilitation, role-play exercises focused on communication and delivering bad news, and narrative reflection. Core content areas included assent and shared decision making, the best interest standard and parental authority, brain death, perceived non-beneficial treatment, medical futility, breadth of parental hope, EOL care, delivering bad news, and moral distress. An OSCE centered on an ethical dilemma was implemented as both a teaching modality and assessment strategy. Facilitators represented a range of disciplines, including pediatrics, ethics, social work, palliative care, medical humanities, law, and psychology. Since participation was determined by rotation schedule and shift assignment, attendance was variable within both the large and small group sessions. Given the monthly frequency and overlapping content across sessions, residents who missed a session were not disadvantaged in their overall curriculum exposure.

### Large group sessions

Six large group sessions were held over one year and open to all residents on day shifts from inpatient rotations or inpatient-based electives. Sessions were approximately 90 min long. Each session presented a real clinical case selected by faculty to cover the session content areas. Facilitated discussion provided residents the opportunity to reflect on the case, explore differing perspectives and examine the moral and ethical considerations arising from the case or from their own experiences. By directing the dialogue toward the tensions, the constraints to action, or the ambiguities of each scenario, the interdisciplinary team helped residents explore the complexities of each case and promoted ethical deliberation. PollEverywhere allowed anonymous real-time responses in all large group sessions.

Session 1 started with the painting “The Doctor” by Sir Luke Fildes as a narrative anchor to open discussion on the breadth of parental hope in EOL care [40]. Subsequent sessions addressed content areas on assent and parental authority, best interests standards, delivering bad news, non-beneficial treatment, and moral distress. Notably, these sessions had overlapping themes and content areas as they arose naturally. The non-beneficial treatment session incorporated excerpts from Ursula K. Le Guin’s “The Ones Who Walk Away from Omelas” and structured written reflection to examine complicity, moral agency, and professional obligation [41]. The delivering bad news session used role play in which faculty and residents interchanged as physician and parent roles, practicing communication strategies and receiving peer and faculty feedback. A psychologist participated in multiple sessions including those on EOL care and moral distress. This was helpful in addressing grief, emotional regulation, and the psychological dimensions of caring for dying children. The final session consisted of the OSCE which provided PGY-3 residents with protected time to apply communication skills and ethical reasoning in a simulated encounter followed by a one-hour debrief.

### Small group sessions

Nine small group sessions were held throughout the year except during transition periods in August 2023 and July/August 2024. These one-hour sessions were held in the PICU with rotating residents (PGY-2 and PGY-3) and PICU fellows. Each session had groups of approximately 8 to 10 learners, and sessions were facilitated by two to three members of the same interprofessional team. Session content areas centered on moral distress, EOL care, brain death, medical futility or non-beneficial treatments, and assent. Similar to the large group sessions, there were overlapping themes. Sessions were based on active cases in the PICU at the time of each session. These small-group discussions provided facilitated ethical inquiry to explore learners' real-time clinical experiences. We utilized Jonsen’s Four Boxes as a structured lens for inquiry into the underlying ethical and moral challenges inherent in the cases [42]. As a narrative reflective exercise, the EOL session incorporated “*The Aquarium,*” an essay by Aleksandar Hemon published in The New Yorker [43]. Hemon’s account of his daughter’s illness and death offered residents a parental lens through which to examine the moral and emotional dimensions of EOL care.

#### Evaluation and feedback (step 6)

Rigorous evaluation explored the curriculum’s impact, sustainability, and feasibility. We implemented Kirkpatrick’s levels of evaluation to assess outcomes [33].

- *Level 1 (Reaction):* Residents provided feedback on curriculum relevance and engagement.
- *Level 2 (Learning):* Pre- and post-curriculum surveys using an instrument with preliminary validity evidence measured changes in cognitive and emotional domains related to moral events.
- *Level 3 (Behavior):* An OSCE based on an ethically and morally challenging case assessed residents’ objective performance.
- *Level 4 (Results):* Recognizing the challenges of demonstrating Level 4 outcomes in educational research, we explored potential indicators of educational impact through pre- and post-curriculum comparative analysis of residents’ written reflections following the OSCE experience. These analyses examined changes in ethical reasoning and communication as early markers that may inform downstream clinical practice. These analyses were not intended as direct measures of Level 4 outcomes.

### Assessment tools

#### Cognitive affective reflection on ethical experiences: moral encounters tool (CARE-MET)

The CARE-MET was developed by the study investigators to measure residents’ reflection on their perceived preparedness on moral-ethical dimensions of care including their comfort with EOL care, confidence in communicating with families of medically complex patients, and their experiences of moral distress. Since no existing instrument adequately addressed our study objectives, we adapted items from previously published tools and developed new items specific to pediatric resident learning needs [20, 28, 44]. The survey included terms such as ‘life-limiting conditions’ (LLCs) and EOL care language to represent clinical settings where moral events are most often encountered. LLCs were defined as medical conditions associated with a shortened life expectancy [45].

##### Validity evidence and reliability

Messick’s validity framework guided collection with preliminary validity evidence established through three sources [46]. Content validity was addressed through expert review for item clarity, relevance, and domain coverage. Revisions were based on consensus. Response process evidence was gathered through two rounds of feedback with eight pediatric residents to verify that items were interpreted as intended. These residents did not participate in the final CARE-MET survey. Internal structure evidence was assessed through exploratory factor analysis. This provided a six-factor solution that explained 63% of total variance and aligned conceptually with our anticipated domains. Internal consistency was good to excellent across five domains (Cronbach's alpha 0.682 to 0.896), with the moral distress domain negatively correlating with the other five domains as anticipated and consistent with its distinct construct. The overall Cronbach's alpha excluding moral distress was 0.781. The final instrument six factors included: factor 1, ‘goals of care decision-making’ (α = 0.896); factor 2, ‘supportive care for families’ (α = 0.750); factor 3, ‘ethical knowledge’ (α = 0.791); factor 4, ‘navigating difficult conversations’ (α = 0.855); factor 5, ‘providing end-of-life care’ (α = 0.803); and factor 6, ‘experiencing moral distress’ (α = 0.682). Given the exploratory nature of this work and the sample size relative to the number of items, these findings should be interpreted as preliminary. Table 1 shows the CARE-MET.

**Table 1:** Cognitive affective reflection on ethical experiences: moral encounters tool (CARE-MET)

**Domain 1: Goals of Care Decision-Making**
Confidence making decisions about withdrawing life sustaining therapies for children with life limited conditions
Confidence making decisions about continuing life sustaining therapies for children with life limiting conditions
Confidence discussing end of life concerns with families
Confidence discussing quality of life concerns with families

**Domain 2: Supportive Care for Families**
I am confident in my ability to comfort families with children of life limiting conditions.^d^
I am confident in comforting a family and their dying child.^d,e^
I am comfortable helping families deal with extreme emotional distress.^d^

**Domain 3: Ethical Knowledge**
What grade, zero (no knowledge) to ten (full knowledge), would you give to your knowledge about end of life care?^a^
What grade, zero (no comfort) to ten (extremely comfortable), would you give to your comfort level when faced with ethical dilemmas or conflicts?^a^

**Domain 4: Navigating Difficult Conversations**
Confidence discussing DNR status with parents of a child with life limiting conditions (i.e. conditions with limited life expectancy)
Confidence delivering bad news.^d^

**Domain 5: Providing End-of-Life Care**
I am comfortable taking care of dying children.^d^
I am comfortable talking to families about the dying process.^d^

**Domain 6: Experiencing Moral Distress**
I find the care of children with life limiting conditions to be a distressing experience.^b,d^
Please rank level of distress (0–4) you feel for the below where 0 is none and 4 is very distressing: Follow the family and patients wishes to continue life support even though I believe it is not in the best interest of the patient and prolong suffering.^c,d^
Please rank level of distress (0–4) you feel for the below where 0 is none and 4 is very distressing: Continue to participate in care for a hopelessly ill person, when no one will make a decision to withdraw support.^c,d^
Please rank level of distress (0–4) you feel for the below where 0 is none and 4 is very distressing: Witness healthcare providers giving “false hope” to patient and family.^c,d^

*DNR* do not resuscitate

^a^Items were rated on a 0–10 scale (0 = no knowledge/comfort, 10 = full knowledge/extremely comfortable)

^b^Items were rated on a 1–6 Likert type scale (never, very rarely, rarely, occasionally, very frequently, always)

^c^Moral distress items were rated on a 0–4 scale (0 = none, 4 = very distressing)

Remaining items were rated on a 1–5 Likert type scale (not at all, not so much, somewhat, fairly well, extremely)

^d^Items that were adapted or modified to fit the needs for pediatric resident learners [20, 28, 44]

##### Timeline of responses

Residents completed the CARE-MET before (T1) and after (T2) curriculum intervention. T1 responses were obtained from residents in August 2023. These residents consisted of PGY-1, PGY-2, and PGY-3 residents. T2 assessments were completed either following 9 months of curriculum intervention to accommodate residents who were graduating or shortly after transition to the subsequent PGY level. To maintain consistency in describing the cohort, their labels refer to their PGY status at the time of participation in the survey. Specifically, our T2 residents consisted of incoming PGY-2 residents (completed survey August-October 2024), graduating PGY-3 residents (completed survey April-June 2024), and incoming PGY-3 residents (completed survey August-October 2024). New incoming PGY-1 residents did not complete the CARE-MET at T2 as they had not yet participated in the curriculum. Since residents were assessed at variable timepoints between 9 and 12 months, paired analysis was selected to account for individual variation in baseline scores and curriculum exposure.

#### An ethically and morally challenging Objective Structured Clinical Examination (OSCE)

##### OSCE creation

Operationalization of a moral encounter within the professionalism milestone requires assessment strategies capable of evaluating empathy and the broader responsibilities of the field including accountability toward patients, society, and the profession [47]. Faculty experts in pediatrics, ethics, humanities, and law developed the case, the assessment tool, and led the debriefing. The case was conducted at the Surgical and Clinical Skills Center at McGovern Medical School.

##### The case

A standardized patient (SP) portrayed a 17‑year‑old girl with newly diagnosed Hodgkin’s lymphoma who was accompanied by her mother (another SP). Four weeks after biopsy, they come to the pediatrician’s office seeking alternatives to chemotherapy. The patient refuses chemotherapy after witnessing her father’s suffering and death from glioblastoma treatment. Her mother supports this decision. The scenario ran for 15 min.

##### Door note

The case synopsis informed the residents that the patient’s cancer had an “80% cure rate,” and that they were the only physician left in the clinic that day. Considering its highly curable nature, respecting the decision of a 17-year-old and her mother conflicted with the trainees’ professional obligation to protect the patient from probable death. While the trainees were not expected to resolve this ethical dilemma or “force” chemotherapy, they were expected to identify the moral and ethical issues inherent to the case, articulate values, communicate concerns, and make permissible recommendations including accepting alternative therapies.

##### SP preparation

SPs were trained over a 2-h period which included learning and practicing appropriate responses based on the case objectives. They were instructed that “Mason Marriot” would never agree to chemotherapy no matter what was said. The SPs were also coached on the performance assessment checklist and were comfortable describing acceptable trainee actions that met the objective requirements.

##### Learner debriefing

The OSCE was video‑streamed for faculty observation which was followed by a one‑hour debrief led by experts. During the debrief, trainees shared impressions while faculty gave feedback on ethical and legal reasoning, addressed behaviors and trends, answered questions, and corrected misconceptions.

#### Evaluative tool

##### Objective measurement

We created a new performance assessment tool consisting of a 30-question checklist to evaluate residents’ demonstration of empathic practice and professional accountability within a morally complex encounter (Table 2). This tool was developed based on existing literature and milestones [30, 31, 47–50]. Items included a dichotomous rating of observed actions (done/not done) along with a section rating empathic behaviors using a 5-point Likert scale ranging from “strongly agree” to “strongly disagree.” At the end of the encounter, SPs also provided verbal feedback to the residents. Both SPs completed the performance assessment once the resident exited the room.

**Table 2:** Performance assessment checklist

Category	Survey Items
Observed behaviors	
	1. Validated the value of parental rights in decision-making in the conversation about best interests for the parent's minor child. (L) 2. Validated minor patient's values and preferences. (L) 3. Paused to allow time for patient and parent to process the information he/she gave rather than fill the silence with facts. (L) 4. Appeared engaged and open with the patient and parent when having this difficult conversation. (L) 5. Warm and friendly (did not appear frustrated, did not come across as combative or interrogative). (L) 6. Empathic during the conversation (reiterated parent/patient concerns to ensure understanding, showed concern for patient's condition and patient's thinking process, voiced fear of outcome without chemotherapy and surgery). (L) 7. Showed concern for both the emotional and physical well-being of the patient. (L) 8. Based on the patient's expressed preferences did not alienate the patient, talk down to the patient, or treat the patient like a child. (L) 9. Valued both patient's and parent's perspectives in this situation. (L) 10. Encouraged patient and patient's parent to ask questions during the encounter. (L) 11. Showed active listening, verbal and non-verbal, during the encounter with patient and patient's parent (nodding, not interrupting patient when he/she was speaking). (L) 12. Used easily understood words, explaining any technical or medical terms in plain language. (L) 13. Told parent and patient that they recommended medical therapies over alternative therapies being truthful, upfront, and frank. (L)
Actions	
	1. Asked to speak to the patient alone. (D) 2. Asked the patient about her/his goals of cancer care. (D) 3. Discussed ways in which the patient's symptoms could be alleviated. (D) 4. Developed an action plan despite conflicting perspectives on treatment plan. (D) 5. Asked the patient how she/he understands and copes with her/his diagnosis. (D) 6. Acknowledged feelings and fears. (D) 7. Asked for the reasons why the patient and parent had reservations/concerns about conventional medical therapies. (D) 8. Asked if the patient would consider both conventional/alternative therapies, or only surgery or only chemotherapy. (D) 9. Explained and emphasized to the patient and parent that the patient's disease has a high chance of survival with conventional medical therapies. (D) 10. Explained "curability" as a benefit that well surpasses the discomfort from the side-effects of chemotherapy. (D) 11. Communicated "death and/or fatality" as the result of not using medical therapies by using words such as "death/dying/fatal." (D) 12. Explained that alternative medical therapies would not cure the cancer. (D) 13. Was willing to work with the patient and her/his family to find solutions for their worries and address the fears they hold against cancer treatment. (D) 14. Was clear that the minor cannot consent to cancer treatment (or decline it, in this case). (D) 15. Stated and explained the standard of parental decision-making (the "best interests" of minor-patient; not what child would want). (D) 16. Prematurely stated that refusing effective cancer treatment is considered child (medical) neglect because of the high curability rate of this type of cancer, and must be reported. (D) 17. Prematurely advised they have a duty to inform TDFPS (Texas Department of Family and Protective Services) on cases of child (medical) neglect. (D)

L, Likert scale rating from “strong agree (5),” “agree (4),” “neutral (3),” “disagree (2)”and “strongly disagree (1).” D, dichotomous rating: 1 = done, 0 = not done. Exception: Survey items 16 and 17 were reverse scored, with 1 for 'not done' and 0 for 'done'

##### Reflective measurement

ollowing the simulated encounter, trainees completed seven reflective prompts that were also developed by study investigators (Table 3). A three-level ethical reasoning framework adapted from Moon et al. and derived from McAlpine’s model of ethical reasoning development guided interpretation of narrative responses [51, 52]. The framework categorized responses according to increasing depth of ethical reasoning, from Level 1 (traditional reasoning: concrete, rule-based) to Level 3 (reflective reasoning: critical, pluralistic, and accountable) (Fig. 1).

**Table 3:** Trainees’ post-encounter self-assessment questionnaire

1	What made this case challenging?
2	Were you conflicted between your professional obligations and your ethical obligations?
3	What do you believe you did well?
4	What would you do differently next time?
5	What is the role of respect for persons, beneficence and nonmaleficence in this situation and which do you believe takes precedence?
6	What options did you identify to pursue with the patient, and what was the rationale for this option?
7	Would you force Mason Marriot to receive surgery and chemotherapy against his/her wishes and why or why not?

All responses were free-text entry

**Fig. 1:**
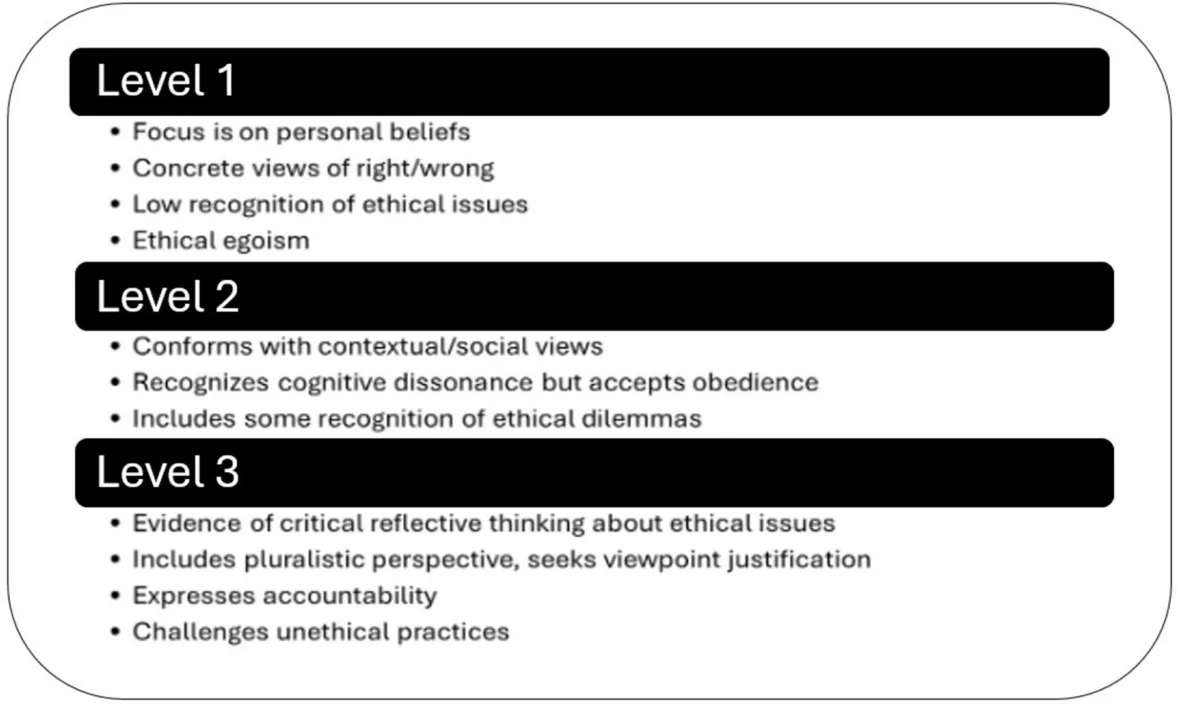
Adapted Ethical Reasoning Tool. Ethical reasoning tool adapted from Moon et al. [51]

##### Piloting

The OSCE was piloted with 32 PGY-1 residents in April 2023 to evaluate the feasibility, clarity, and relevance of the case and assessment tool. Residents and SPs completed separate debriefs to confirm functionality. Pilot data were used solely for refinement purposes and pilot participants were excluded from all comparative analyses. Following piloting, two independent PGY-3 cohorts completed the OSCE in June 2023 (T1) and June 2024 (T2) (Fig. 2).

**Fig. 2:**
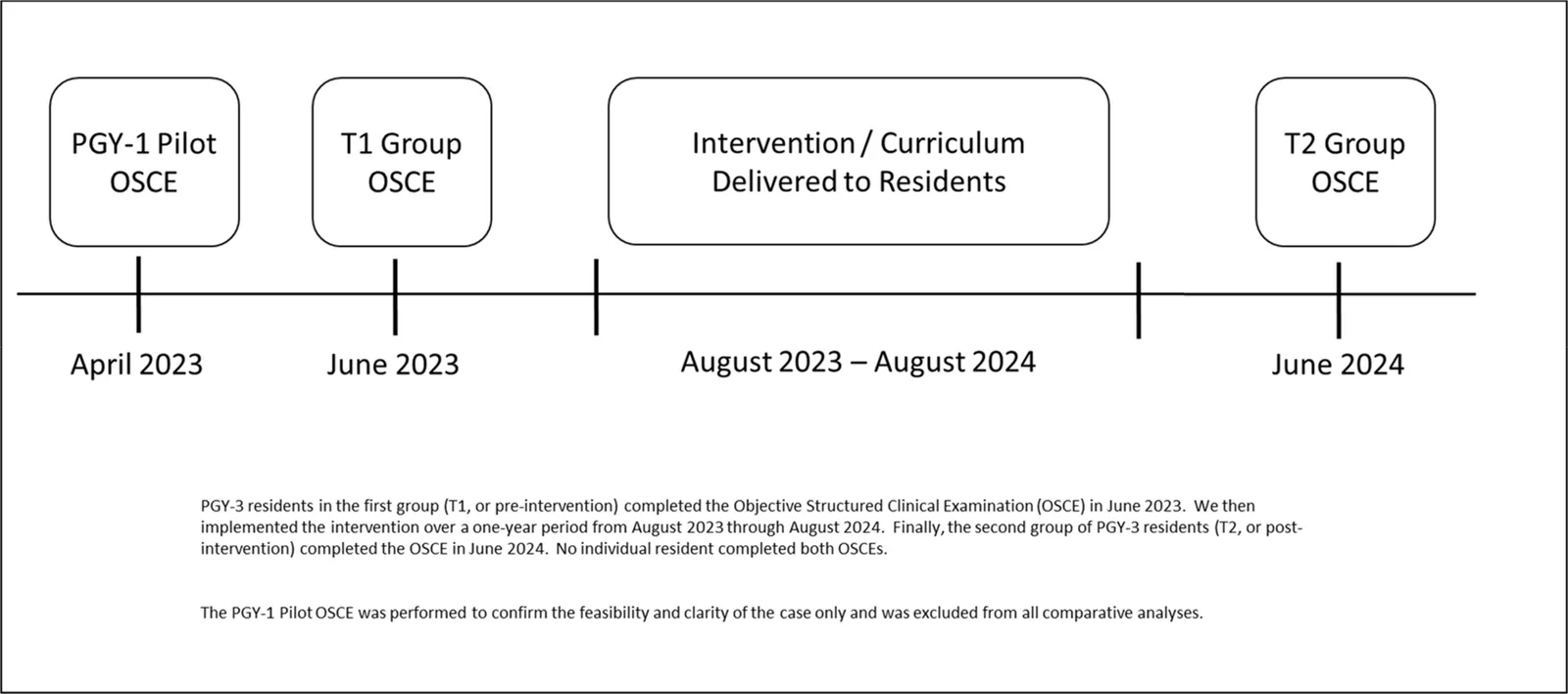
Timeline of OSCE for PGY-3 at T1 vs PGY-3 at T-2

#### Data analysis

##### OSCE analysis

Fisher’s exact test compared individual checklist items between cohorts. Items were also grouped a priori into two domains based on expert consensus: observed behaviors reflecting empathic practice, and action items reflecting professional accountability. Domain level comparisons used averaged Likert scores (1–5) for empathic practice behaviors and summed scores for binary accountability action items. We analyzed comparisons between cohorts in both domains using Mann–Whitney U tests. Box plots provided visual representation of distributions at T1 and T2.

Trainee reflective writing underwent content analysis conducted by three members of the research team who did not facilitate the OSCE (AM, MO, and AM). These researchers graded reflections blinded to T1 or T2 cohorts to minimize bias. All reviewers independently coded the data using an a priori theoretical framework of ethical reasoning [51, 52]. When participants did not demonstrate a response corresponding to a specific ethical reasoning component, that component was coded as 0. This value indicated the absence of that component rather than a lower level of reasoning. Discrepancies were resolved using a consensus approach, with agreement defined as concurrence by at least two of the three reviewers. Chi-square tests compared the overall distribution of ethical reasoning levels between T1 and T2 cohorts.

##### CARE-MET analysis

Mann–Whitney U tests compared ordinal variables between two groups whereas comparisons involving three or more groups used Kruskal–Wallis tests. When Kruskal–Wallis results were statistically significant, post hoc pairwise comparisons were performed using the Dwass–Steel–Crichtlow–Fligner (DSCF) method to identify specific group differences. Paired analyses of participants at T1 and T2 used Wilcoxon Rank Sum Test with effect sizes calculated using Cohen’s *d*.

Residents’ evaluative responses on the perceived value and key takeaways from the curriculum underwent thematic analysis by two independent coders (MO, AM) who reached consensus.

A two-sided *p*-value of < 0.05 was considered statistically significant. All statistical analyses used statistical software R 4.5.0.

## Results

### Kirkpatrick level 1 (reaction)

Of 81 eligible pediatric residents, 65 (80%) completed the CARE-MET at T1. Of 57 eligible residents at T2, 52 (91%) completed the survey. Of those 52, the majority reported attending 2–3 large group sessions (67%), 18% attended one large group session, and 15% attended four or more large group sessions. Attendance was highest among PGY-3 residents with 20 out of 27 (74%) PGY-3 respondents reporting attending 2–3 sessions and 7 out of 27 (26%) attending 4 or more sessions.

Overall, 97% (64/65) of residents found the curriculum valuable, and all PGY-3 residents (*n* = 12 at T1 and *n* = 14 at T2) found the OSCE valuable. Qualitative feedback responses revealed four dominant themes in what residents found meaningful: (1) professional growth and confidence (2) the use of real clinical cases as the basis for discussion, (3) the opportunity to hear diverse perspectives from peers and faculty, and (4) the space to process emotional responses to morally distressing clinical experiences. Resident responses included: “It is okay to have moral distress, it keeps us human.” Residents also identified the OSCE as uniquely valuable: “The SP session helped point out deficiencies in direct patient care which would not otherwise have been brought to my attention during traditional roundtable discussions” (Table 4).

**Table 4:** Residents’ reported values and takeaways from the curriculum

What was valuable	Key takeaway
Professional growth and confidence	
Awareness of ethics principles and approaches to ethically challenging or ambiguous scenarios	Essential but complex aspect in decision-making for all patients esp those with life-limiting conditions
I feel much more confident when talking with families about end-of-life care even if I may never fully feel comfortable in that situation	These ethics sessions provided teaching that is not provided in our training. It made me feel more confident and comfortable with challenging ethical decision making
Learning how to communicate with families effecrively*	Be comfortable sitting in discomfort
Value of real cases and reflection	
The SP session helped point out deficiencies in direct patient care which would not otherwise have been brought to my attention during traditional roundtable discussions	There are times when we as providers have to engage in patient care that may feel inappropriate to us in order to provide parents with autonomy over their child's care
I appreciated that these were all real patient cases that encouraged us to reflect on our patient care practices and dilemmas that we encounter on a day to day basis	That there is no right or wrong answer but that the least we could do is carry out plans that the entire team (including parents) agreed upon
Exposure to diverse perspectives	
Being presented with different scenarios and view points helped open up my mind to other perspectives so that I am not so instantly closed minded when hearing a different opinion	Ethics can sometimes be a very gray area. The only way to clear discrepancies is to spend time getting to know your patients to make the best decision. There is no one size fits all policy
Processing experiences, remembering the family/parents' side of the story and their experience, having Dr. Major-Kinkaid talk, getting outside of myself/my experience with a certain patient	There are so many aspects to a patient's care (from things the family is dealing with/dealt with, countless behind the scenes conversations happening with other providers, etc.); I can feel better and perform better when I talk w/people who understand what I've seen; our palliative care team is so valuable
Ethics impacts every medical decision	Making sure to be aware of my personal biases and keeping the patient and family in mind for medical decision making
Safe space for emotional processing	
The experience and healing process with each learning case and opportunity	Sometimes as providers to children we have to be comfortable with a certain level of moral distress and we do not have to reconcile this moral distress on our own
Knowing that my peers had same feelings of distress	Its okay to admit you don't* know
Open discussion of difficult topics we probably neglect to think about	
Self-reflection and bias awareness	
Becoming more self aware of my own biases and having more comprehensive thoughts in regards to discussions with family	I think they have helped me become much more aware of how to frame difficult conversations with families
Facilitated safe and open discussion on various real and difficult scenarios that allowed me to hear various perspectives and help inform my own perspective	Serious consideration of the ethical implications of a clinical scenario need to be addressed early on as a team and with the family in order to try to mitigate as much moral distress as possible
Navigating moral distress	
Learning to distinguish between moral distress and ethical dilemmas, offering perspectives we haven't considered	Even though we disagree and feel moral distress, our role is not to make the decisions, it is to counsel families and empower them to make their own decisions. Our duty is to treat the patient while taking into account that the family is with the patient every day and we should trust them to decide. It is also our duty to present an accurate picture so the family can be best informed when making their decisions

Themes Ordered from Most Common to Least Common

Post-curriculum feedback responses organized thematically (*N* = 40 residents, PGY-1 through PGY-3)

*PGY* post-graduate year, *SP* Standardized patient

^*^All responses verbatim as written by residents including spelling and punctuation

### Kirkpatrick level 2 (learning)

#### Pre- vs. post-curriculum comparisons

The gender, race, and ethnicity distribution of both cohorts mirrored that of the overall pediatric residency program and there were no significant differences between T1 and T2 demographics.

##### Paired analysis

A paired analysis was conducted on 37 participants who completed both T1 and T2 assessments. Scores demonstrated significant improvement at T2 compared to T1 in five of the six factor domains with large effect sizes (Cohen’s *d* > 0.7). Experiencing moral distress did not change significantly with a small Cohen’s *d* = 0.307 (Fig. 3).

**Fig. 3:**
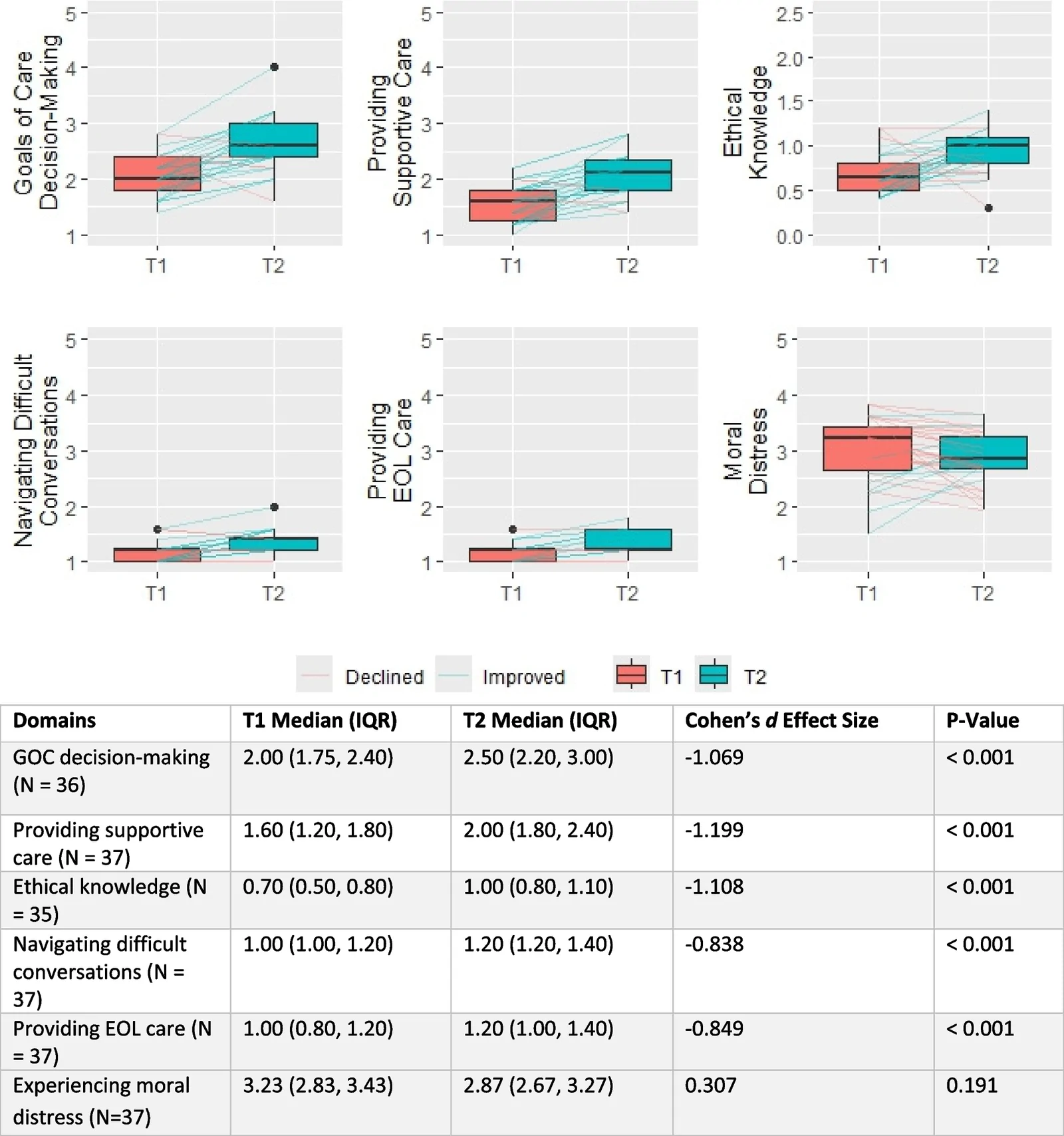
Paired Analysis Comparing Pre- and Post-Curriculum Scores of Matched Participants. IQR, interquartile range; T1, timepoint for pre-curriculum intervention; T2, timepoint for post-curriculum intervention; GOC, goals of care; EOL, end-of-life. Data are presented as median (interquartile range). Box plots illustrate comparisons across six factor domains between T1 and T2 among matched participants who completed both survey administrations. Between timepoint differences were analyzed using the Mann–Whitney U test. Statistical significance was defined as *p* < 0.05. Effect sizes were calculated using Cohen’s *d*. Domains on goals of care decision making, providing supportive care, navigating difficult conversations were scored on a Likert scale 1–5. Domain on ethical knowledge was scored on a range from 1–10. Domain on experiencing moral distress was scored on a Likert scale 1–5. The levels of moral distress were rescaled from 0–4 to 1–5. One item from the moral distress domain originally rated on a 1–6 frequency scale was rescaled to a 1–5 scale using a proportional linear transformation to preserve relative spacing across response options. On the moral distress domain, higher levels were indicative of higher distress

##### Gender differences

T1 analysis by gender indicated that male residents reported greater confidence in factor 1 (goals of care decision-making), factor 4 (navigating difficult conversations) as well as in factor 5 (providing end-of-life care). At T2, no significant gender differences were observed across any of the six factor domains.

Qualitative responses further illustrated residents' learning at both affective and cognitive levels. Resident description of the curriculum clustered into three domains: cognitive (e.g., 'thought-provoking,' 'enlightening,' 'reflective'), affective (e.g., 'therapeutic,' 'heart-breaking,' 'uncomfortable'), and evaluative (e.g., 'invaluable,' 'insightful,' 'informative’).

### Kirkpatrick level 3 (behavior)

Pre- and post-OSCE comparisons provided data to evaluate changes in behavior. There were 12 out of 18 (67%) and 14 out of 18 (77%) PGY-3 residents in the T1 and T2 cohort respectively.

#### Objective outcomes

When comparing individual observed behaviors and action items, one item showed a statistically significant difference. At T2, 36% (5/14) of PGY-3 residents communicated risk of “death/fatality” without chemotherapy which was up from 0% at T1 (p = 0.024).

Medians and interquartile ranges for T1 and T2 are presented in Fig. 4 as two domains: averaged empathic behavior scores and summed accountability action items. Residents consistently demonstrated empathic practices in both cohorts. Although no statistically significant differences were observed between T1 and T2 for action items, both cohorts demonstrated lower overall scores relative to empathic behaviors.

**Fig. 4:**
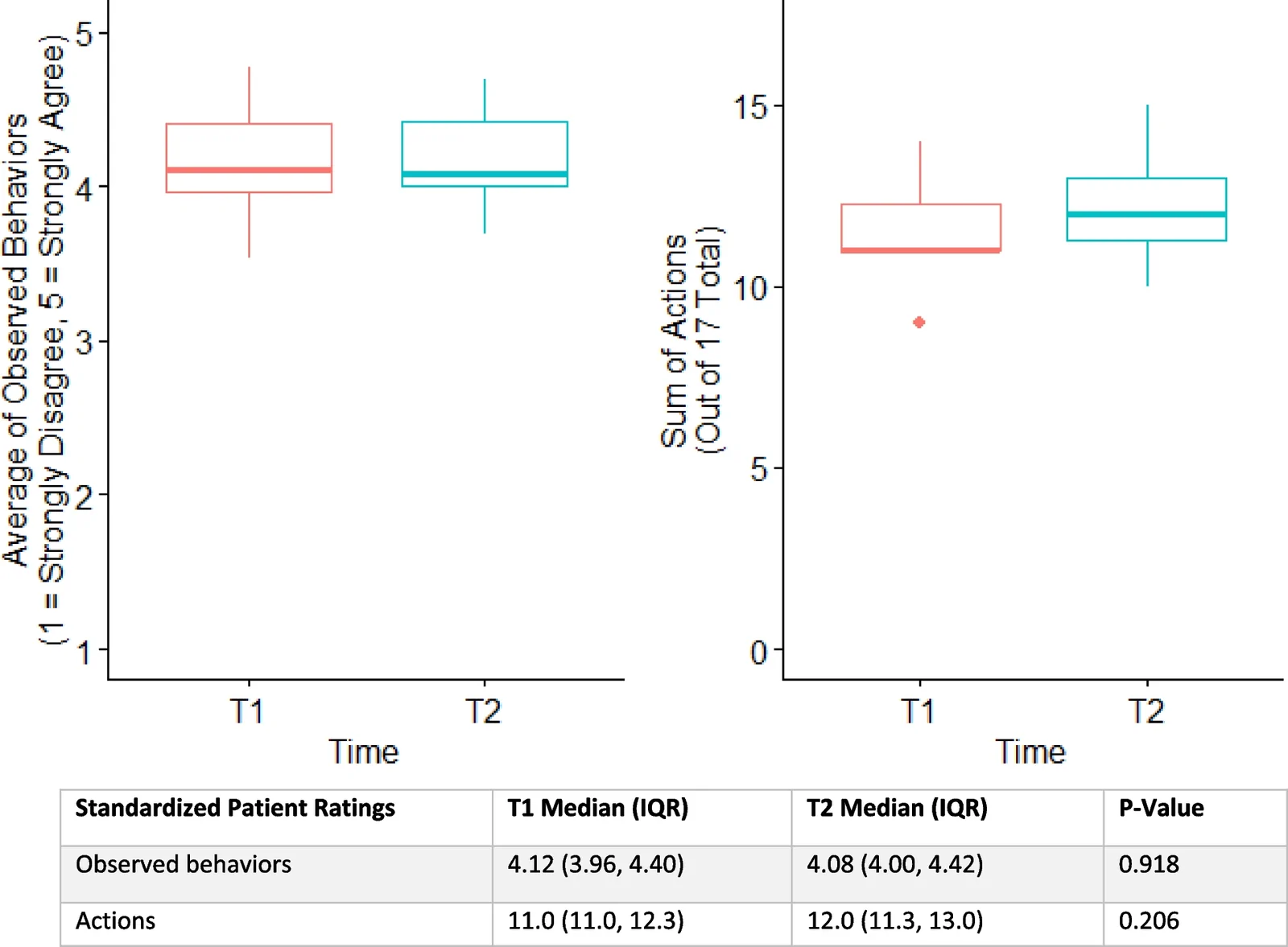
Observed Behaviors and Actions in a Morally and Ethically Challenging Case. T1, timepoint before curriculum intervention; T2, timepoint after curriculum intervention; IQR, interquartile range. Data are presented as median (interquartile range). Box plots illustrate comparisons between PGY-3 residents at T1 versus T2 across two assessment categories: Likert-scaled items (observed behavior) and binary items (actions). Action items were summed to generate a total action score for each participant (maximum score = 17). Likert scores (1–5) were averaged. Between group differences were evaluated using Mann Whitney U test. Statistical significance was defined as *p* < 0.05

#### Reflective writing outcomes

A greater proportion of residents scored the highest Level (3) at T2 compared with T1 (Table 5).

**Table 5:** Overall ethical reasoning level of distribution at T1 vs T2 m

Reasoning Level	T1 (*n* = 96 observations)	T2 (*n* = 112 observations)	*P*-value
Level 3: Reflective	6 (6.2%)	22 (19.6%)	0.004
Level 2: Traditional/Reflective	27 (28.1%)	28 (25.0%)	0.610
Level 1: Traditional	46 (47.9%)	40 (35.7%)	0.075
Level 0: Not addressed	17 (17.7%)	22 (19.6%)	0.722

T1, timepoint before curriculum intervention; T2, timepoint after curriculum intervention, n, number of observations

There were 8 distinct ethical reasoning components scored with an ordinal scale ranging from 1 (lowest score) to 3 (highest score)

n, represents the total number of observations at T1 (12 residents × 8 components = 96 observations) and T2 (14 residents × 8 components = 112 observations)

Chi-square test, *P* value significant < 0.05

Figure 5 illustrates the distribution of ethical reasoning scores in all eight components at T1 and T2. At T1, median scores for most components fell in the lower to mid range. By T2, medians shifted towards higher scores across several components.

**Fig. 5:**
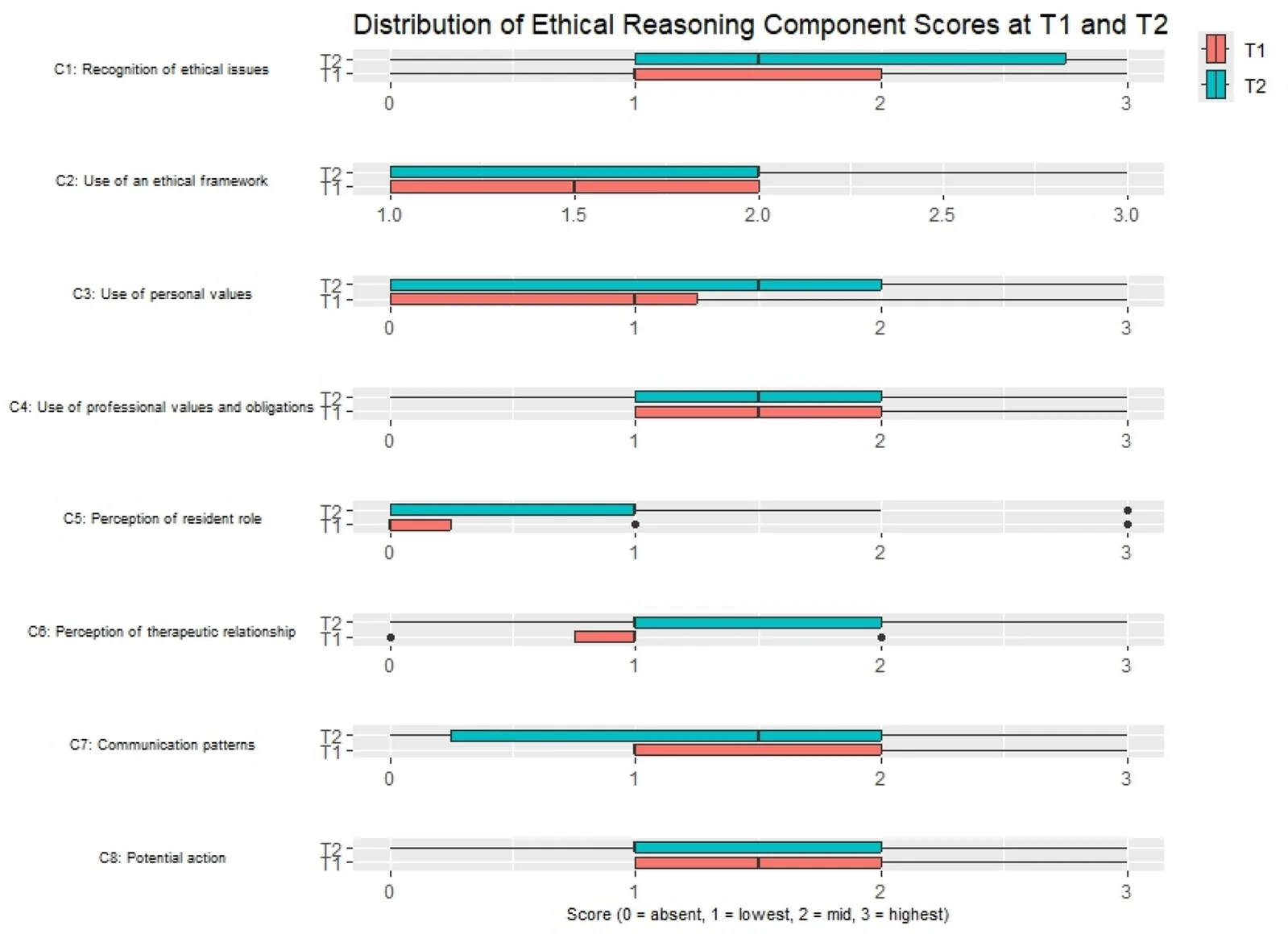
ox Plot Demonstrating Distribution of Competency Scores Before (T1) and After (T2) the Intervention. Horizontal box plots display an overlay of the distribution of scores for each of the eight components at pre-intervention (T1) and post-intervention (T2). Each row on the y axis corresponds to one of the 8 ethical components assessed. Each marker on the x-axis represents the participant’s score on a 1–3 ordinal scale (1 = lowest, 3 = highest). A score of 0 was assigned when no response was observed that corresponded to a given ethical reasoning component. For each box plot, the central line represents the median, and the left and right edges of the box represent the 25th and 75th percentiles (interquartile range). Whiskers extend to the most extreme values not classified as outliers. Individual outliers are shown as dots

Tables 6 and 7 illustrate the resident reflections as they move from level 1 (traditional) to level 3 (reflective) ethical reasoning.

**Table 6:** Illustrative resident reflections by ethical reasoning component: C1–C4 (cognitive and values based components)

Component & Question	Level	T1 (Pre-curriculum)	T2 (Post-curriculum)	What shifts between levels
C1: Recognition of ethical issues				
What made this case challenging?	Level 3	"This case was challenging because the patient was suffering from a disease that had a good likelihood of cure with treatment, and yet, the family still did not want to pursue treatment." Evaluatee 5754	"Having to handle a different viewpoint that was contradictory to my own views. It was also not something I would recommend to anyone so it was hard being respectful but also being honest." Evaluatee 6310	Identifies the core ethical tension: the conflict between clinical obligation and patient/family values, and the difficulty of holding both with integrity
C2: Use of an ethical framework				
What is the role of respect for persons, beneficence and nonmaleficence, and which takes precedence?	Level 1	"Nonmaleficence comes first because you would never harm a patient, respect comes next because patients will never listen to anything you say if they do not feel respected" Evaluatee 9045	"Respect for the person takes precedence and then non-maleficience" Evaluatee 8887	Names one or two principles without applying them to the specifics of the case. No framework used to analyze competing obligations or justify prioritization
What is the role of respect for persons, beneficence and nonmaleficence, and which takes precedence?	Level 3	*No responses at this level*	"I think they all go hand in hand. I think respect for persons/autonomy is incredibly important and lays the foundation for future interactions and trust in their medical provider. It's important to understand that non maleficence and beneficence also applies to non-science things like—are we doing harm to this patient mentally by forcing him/her to undergo a treatment he/her doesn't want to?" Evaluatee 8894	Uses the framework to organize competing obligations as distinct professional duties, justifies prioritization with reasoning grounded in the specific case, and acknowledges moral ambiguity
C3: Use of personal values				
Were you conflicted between your professional and ethical obligations?	Level 1	"Yes. Personally, I wanted her to undergo the treatments" Evaluatee 9045 "The patient and mother had already made up their mind about their plan which was not in line with my ethical thought process." Evaluatee 9601	"If I had "my way", I would obtain a court order and start the patient on curative therapy instead of allowing her mother to refuse allopathic treatment. Ethically I was obligated to understand the patient's concerns and give the family space to refuse therapy." Evaluatee 9070	States personal values or preferences without examining them or identifying the conflict they create. Values are asserted as justification rather than analyzed as one element within a larger frame
Were you conflicted between your professional and ethical obligations?	Level 3	"Not at all. Patient autonomy, especially for a 17-year-old, drives patient care. This family had a clear understanding of the disease, treatment options, and what would happen if treatment was not pursued. My professional obligation was to care for the patient as best as I could and as much as she would allow while my ethical obligation was to make sure the family understood what lack of treatment meant. In the end, those obligations led to the same point—supporting them how they wanted to be supported." Evaluatee 5754	"I did feel conflicted because I would choose to make a different decision than that of the patient's. However, I believe that every one should have their own say about what they want in their life and to respect their wishes as much as possible (within legal limits—although laws can also be changed)." Evaluatee 8894	Identifies personal values as one component of analysis and includes alternative viewpoints within the response. Demonstrates capacity to detach from personal preference in service of a larger ethical frame
C4: Use of professional values and obligations				
Were you conflicted between your professional and ethical obligations?	Level 3	"Not at all. Patient autonomy, especially for a 17-year-old, drives patient care. This family had a clear understanding of the disease, treatment options, and what would happen if treatment was not pursued. My professional obligation was to care for the patient as best as I could and as much as she would allow while my ethical obligation was to make sure the family understood what lack of treatment meant. In the end, those obligations led to the same point—supporting them how they wanted to be supported." Evaluatee 5754	"Somewhat – i really think conversations like these happen over the long-term and there are aspects that are completely understandable. if down the line they formally decided against any chemo or surgery i think it would be much harder, but by that point we would have had multiple conversations and i could probably understand more about the daughter/mom dynamic as well, have one-on-one conversations with each of them, and get a better idea that they understand the repercussions and the decisions they could potentially be making" Evaluatee 8905	Names specific professional obligations, justifies how they were applied, and situates the analysis within the longitudinal relational context of care. Demonstrates recognition that professional obligations and patient autonomy can converge rather than conflict
C4: Use of professional values and obligations				
Would you force Mason Marriot to receive surgery and chemotherapy, and why or why not?	Level 1	*“*Of course not. She is 17, has come to her own conclusions, and has a mother who agrees with the patient.” Evaluatee 9045	“No; I believe that every patient still ultimately should reserve the right to make their own informed medical decisions” Evaluatee 8899	States professional values as rules or simple assertions without examining competing obligations or contextualizing them within the case. No justification offered for how professional values should be applied
Would you force Mason Marriot to receive surgery and chemotherapy, and why or why not?	Level 3	*No responses at this level for this specific question*	"No I would not. If she and her mother are fully informed about the decision, then I would not be respecting her as a person. By forcing her through treatment, I strip her of her autonomy. She has valid reasons for her decision, and I would not want to further harm her by forcing her to do things she does not want to do. I feel by stripping away autonomy, I am stripping away a person's humanity. And since I became a doctor to treat humans, I feel like I am failing at my profession." Evaluatee 6310	Names professional obligations, justifies the choice, and connects professional identity to the ethical decision

Components C1–C4 address how residents recognize ethical issues and apply ethical, personal, and professional frameworks

All quotes verbatim and unedited. T1 = pre-curriculum cohort (June 2023, *n* = 12); T2 = post-curriculum cohort (June 2024, *n* = 14). Level 1 = Traditional reasoning; Level 3 = Reflective. 'No responses at this level' = component not reached at that level for that timepoint. Scores reflect blinded consensus coding by three independent reviewers using an ethical reasoning framework [50]

**Table 7:** Illustrative resident reflections by ethical reasoning component: C5–C8 (relational and behavioral components)

Component & Question	Level	T1 (Pre-curriculum)	T2 (Post-curriculum)	What shifts between levels
C5 and C6: Perception of resident role and therapeutic relationship				
What do you believe you did well?	Level 1	"Not being judgemental and attempting to work with the family." Evaluatee 9601 “I believe I heard the families perspective well.” Evaluatee 9602	"Connecting with patient and parent and making them feel respected" Evaluatee 8897 "I made the patient feel comfortable and empowered to remain active in her treatment." Evaluatee 8896	Describes a relational behavior where the residents’ actions are reported but not framed within a sense of professional responsibility or purpose
What do you believe you did well?	Level 3	"I believe I included both the patient and her mother in the conversation while firmly stating that I recommended pursuing conventional treatment, but that I would walk with them however they wanted me to during this journey (specifically with alternative treatments). I did not dismiss either the patient or her mother, but left the communication channel open." Evaluatee 5754	"Listened well, engaged genuinely, got the reasoning behind why they didn't want to do chemo/surgery out pretty quickly, made actionable steps" Evaluatee 8905 “I felt like I was respectful and presented information in a clear and respectful manner that honors the dignity of patients.” Evaluatee 6310	Articulates a clear role definition: advocate for informed decision-making while maintaining the therapeutic relationship and leaving the communication channel open
C7: Communication patterns				
What would you do differently next time?	Level 1	"Have more knowledge on legal ramifications of refusing life saving treatment in a child." Evaluatee 7712 "Look up if a 17 year old has adult rights in the state of Texas" Evaluatee 9045	"Be more direct about letting her know the consequences of not undergoing chemotherapy" Evaluatee 8897 "Be more open to proceeding with alternative medicine route sooner in the conversation" Evaluatee 8899	Identifies knowledge gaps or surface level adjustments without reflecting on communication patterns
What would you do differently next time?	Level 3	"Would have liked to talk about typical goals of patients/families who choose alternative therapies and contrast them to goals of the patient, which she emphasized that she would like to return to normalcy." Evaluatee 7711	"More eye contact with the actual patient and also be more direct in saying words like "death and dying" especially when the patient themselves has used similar language." Evaluatee 6310 "Ask more specific questions about what she saw with her dad and how much time it bought him, what she learned in therapy, larger belief systems (faith, etc.)" Evaluatee 8905 "I would spend more time exploring experience with father in hospital to make deeper connection" Evaluatee 8893	Identifies strategies that move beyond traditional communication patterns. Resident responses are meeting the patient in their own language, exploring narrative and belief systems, and acknowledging difficult realities
C8: Potential action				
What options did you identify to pursue with the patient?	Level 1	"We discussed her goal setting and let that be the path that leads to whatever treatment options she has" Evaluatee 7711 "I offered to provide information on both routes of therapy" Evaluatee 9046	"Talking with the oncologist and reviewing their options" Evaluatee 6325 "Further workup of SOB; Iron pills for Hgb" Evaluatee 8887	Identifies a single or vague action without rationale. No evidence of a strategy that integrates competing care needs or demonstrates a clear plan for follow-up
What options did you identify to pursue with the patient?	Level 3	"I presented the option of researching alternative therapies for symptom management; my rationale for this option was that the family, specifically the patient, had considered all options and selected alternative therapies as the best personal choice for her and her family. Their minds were made up so any other course of action would have been futile. I left an opening that my professional recommendation would be to pursue conventional therapy, which she was free to do at any time, but in the absence of pursuing conventional therapy, I would help to manage her symptoms as best as I could." Evaluatee 5754	"Talk with pedi oncology: they have expert insight into the specific chemo and surgery she would receive if they went that route, probably have experience or at least exposure to patients who seek alternative treatments, and they often have support staff specific to their situation; address fatigue and SOB: if anything else is contributing that we can address medically it will help build further rapport and improve her quality of life immediately; follow up quickly; connect to support groups: help them see kids who have received treatment and done exceptionally well" Evaluatee 8905	Presents a comprehensive strategy with rationale for each action, integrates multiple care pathways simultaneously, and demonstrates a clear desire to implement change while keeping the therapeutic relationship intact

Components C5–C8 address how residents perceive their role and therapeutic relationship, understand their communication patterns, and identify potential actions within the clinical encounter

All quotes verbatim and unedited. T1 = pre-curriculum cohort (June 2023, *n* = 12); T2 = post-curriculum cohort (June 2024, *n* = 14). Level 1 = Traditional reasoning; Level 3 = Reflective. 'No responses at this level' = component not reached at that level for that timepoint. Scores reflect blinded consensus coding by three independent reviewers using an ethical reasoning framework [50]

## Discussion

Few studies have examined how pediatric residents cognitively and emotionally experience moral encounters, how such experiences shape professional agency, or how to systematically measure growth in these domains [14]. Without a structured framework, residents may struggle to reconcile ethical reasoning and professional responsibility within clinical practice. Our pilot curriculum prioritized areas that most frequently generate uncertainty, moral distress, and tension in pediatric residents. We grounded design in Kern’s six-step framework and residents’ lived experience to ensure the curriculum was needs driven and structured [32].

### Curriculum assessment tools

Despite their potential, simulated patient encounters emphasizing professionalism and ethical dilemmas are sparsely utilized in pediatrics [31]. Our OSCE emphasized applied ethical reasoning, highlighting the moral, legal and ethical dimensions of patient care. The CARE-MET assessed learners' self-efficacy in multiple domains related to moral encounters including goals of care decision-making, supportive care for families, ethical knowledge, navigating difficult conversations, providing end-of-life care and experiencing moral distress. The two tools provided complementary perspectives on learner growth and provided pertinent insights into observed performance and perceived self-efficacy.

### Curriculum outcomes

The intervention’s educational value was reflected in the consistently positive responses reported by residents at every training level. Residents described the sessions using both distressing and affirming language. They characterized them as simultaneously “uncomfortable,” and “invaluable,” or “heart-breaking,” and “empowering.” The coexistence of negative and positive descriptors is reflective of the curriculum’s design to surface moral distress as a common feature of ethical practice.

The curriculum’s first goal was to provide residents with the skills to recognize and navigate moral encounters in clinical practice. Paired analysis of CARE-MET demonstrated significant improvements in all domains except experiencing moral distress. The lack of reduction in moral distress was an anticipated result of a curriculum that aimed to build recognition and normalization of distress rather than resolve it. Changes at T2 compared to T1 in the CARE-MET were characterized by a large Cohen’s *d* which suggests the intervention likely contributed to the magnitude of growth. Gender differences observed at baseline were also eliminated post-curriculum. This finding is of interest given prior literature documenting gender disparities in comfort with EOL discussions though the single-center design limits generalizability of this result [20]. Further highlighting achievement of our first goal was the significant three-fold increase in Level 3 reasoning in the OSCE reflective writing at T2. Residents moved from traditional rule-based ethical reasoning toward more pluralistic, reflective reasoning at T2. This shift toward richer and more nuanced reflections suggests that curricular exposure supported resident advancement in the cognitive dimensions required to navigate moral encounters.

The curriculum’s second goal was to guide trainees in recognizing and analyzing ethical principles while providing the educational infrastructure for values clarification. One specific goal included the ability to differentiate between concepts of moral distress and ethical dilemmas. Resident feedback provided insight into how the curriculum added value to their professional and emotional development, as well as their overall engagement with the material. The curriculum’s emphasis on psychological safety provided a necessary foundation for such a development [53, 54]. One participant identified an improved capacity to distinguish between moral distress and ethical dilemmas while another reported a subjective increase in confidence during EOL discussions stating, “I feel much more confident when talking to families about end of life care even if I may never fully feel comfortable in that situation.” Residents also explored the clarification of their values through interconnectedness with their peers. Residents provided feedback such as, “there is always more than one answer” and “being presented with different scenarios and viewpoints helped open up my mind to other perspectives so that I am not instantly close minded when hearing a different opinion.” These responses illustrate a growing capacity to frame emotional experiences as opportunities for professional learning.

The curriculum’s third goal was to emphasize moral sensitivity through recognition of empathy as a core dimension of care. In the post-curriculum evaluation, residents described the curriculum using cognitive and affective descriptors reflective of moral sensitivity. While the OSCE results complemented this finding, they also highlighted a less linear trajectory in observed behavior. Consistently high empathic practice scores at both timepoints suggest that relational and communicative behaviors were already relatively established among third-year residents. The most notable behavioral shift was that T2 residents more frequently communicated death as a potential consequence of treatment refusal, even when navigating a complex moral encounter. Physician trainees commonly lack the formal training and tools to disclose emotionally difficult truths, and EOL communication skills are frequently underdeveloped in graduate medical education [12, 13, 55, 56]. This shift suggests that curricular exposure may have supported residents in overcoming the tendency to use vague or euphemistic language when discussing critical outcomes. During the OSCE debrief, T2 resident reflections also suggested increased awareness of the physician’s duty to voice recommendations clearly, even if decisional authority rests with parents or caregivers.

### Ongoing challenges

Beyond the primary curriculum outcomes, two recurring patterns emerged from the OSCE as secondary findings for future refinement: uncertainty about legal obligations in pediatric decision-making, and hesitancy in assuming professional accountability in morally distressing encounters. The OSCE revealed that trainees’ skills fell into two categories: learned behaviors and learned medical professionalism competencies. Learned behaviors are more intuitive, such as showing compassion during a difficult conversation, while learned medical professionalism competencies, such as structured ethical and moral reasoning, require formal instruction to develop [57]. Both cohorts demonstrated persistent gaps in knowledge of the professional, legal, and ethical obligations necessary in more complex situations. This hesitancy is not surprising as residents occupy a constrained role within the medical hierarchy and can frequently become bystanders to complex moral events.

#### Limitations

This study has several limitations. First, as a single-center study, our findings may not be generalizable to other institutions or residency programs. Second, the OSCE component had a limited sample size composed of third-year residents. This limited sample size was primarily due to the associated costs of simulation encounters and challenges in scheduling additional simulation sessions during protected resident time. These challenges restricted the OSCE to a single encounter. End-of-year timing and conflicting commitments by soon-to-be graduating residents further limited participation. The OSCE was also designed primarily as a formative learning and evaluation tool. Although we did not perform inter-rater reliability testing, there were multiple measures to ensure integrity of our evaluation process. We had 2 SPs per trainee and required mutual agreement by both on resident actions and observed behaviors. The performance assessment tool primarily consisted of dichotomous ratings. Additionally, faculty members monitored video recordings and confirmed consistency.

The OSCE involved highly controversial content. This was by design and allowed us to (1) examine residents’ approach to the morally challenging encounter and (2) identify strengths and deficiencies of residents’ ethical reasoning. Given the multiple challenging aspects of the case, we were surprised that the age of maturity became a primary point of contention among residents. While many residents did not recognize the patient as a minor requiring parental consent, they clarified during the debrief session that their ethical dilemma would remain regardless of age, unless the patient was a very young child. One resident articulated a broader concern that contemporary medical practice now spans a spectrum from “paternalistic practice to concierge medicine,” making it difficult to discern the resident’s appropriate role.

While the SP encounter was brief, it was followed by a resident self-assessment questionnaire and up to an hour-long debriefing session. In the debrief, most residents admitted to being protective of the physician–patient relationship. Residents confirmed that they prioritized relational continuity over transparency regarding prognosis. These reflections revealed the complexity of prognostic discussions and the tension residents experience between building a longitudinal therapeutic relationship and providing transparent communication about prognosis.

Although the CARE-MET demonstrates internal consistency, it is a self-report measure and is therefore subject to response bias and social desirability effects. As the tool is at an early stage of validation, findings from this tool should be interpreted with caution. We acknowledge that maturation and time-in-training may have influenced residents’ development. However, the magnitude of effect size and patterns of the change observed are more consistent with the effects of a structured educational intervention than with typical progression through residency. Additionally, while the curriculum emphasized experiential and reflective learning, its implementation was limited to one institution which makes it difficult to determine long-term retention or transferability across residency stages. These limitations suggest that future studies should employ multi-center samples, incorporate formal inter-rater reliability analyses, and include longitudinal follow-up to assess the sustained impact of the intervention.

## Conclusion

Our curriculum offers early evidence that structured, case-based education on moral encounters can support residents’ growth in perceived self-efficacy, and ability to engage honestly with complex ethical challenges. Pairing curriculum design with complementary assessment tools allowed us to examine learner growth across multiple levels. Our findings suggest that while residents may be well equipped to respond empathically, they often remain insufficiently prepared to assume agency and navigate the moral, ethical, and legal dimensions of complex pediatric care. Professional accountability within morally fraught encounters remains a developmental frontier for residents, and a one year educational intervention is unlikely to fully address this challenge. The identified gaps of ethical and professional knowledge among third-year pediatric residents are likely systemic across pediatric training programs. Literature consistently documents inadequate ethics preparation in graduate medical education [3, 4, 12–14, 58]. Incorporating practical education on moral encounters into residency curricula may better prepare residents for the complexities of contemporary pediatric practice. The development of tools such as the CARE-MET represents an early but important step toward measuring growth in the cognitive, and affective dimensions of how residents navigate moral events in clinical practice. Ongoing psychometric validation will further strengthen the ability to evaluate these essential outcomes in medical trainees.

## Data Availability

The datasets generated during and/or analyzed during the current study are available from the corresponding author on request.

## Abbreviations

ACGME: Accreditation Council of Graduate Medical Education
AAP: American Academy of Pediatrics
AM for Amanda McCarthy or Aymen Mirza, as applicable, and MO for Margarita Ortiz: Author initials
CARE-MET: Cognitive Affective Reflection on Ethical Experiences: Moral Encounters Tool
DSCF: Dwass–Steel–Crichtlow–Fligner post hoc test
EOL: End-of-life
HSC-MS: Health Science Center–Medical School
IQR: Interquartile range
IRB: Institutional Review Board
LLC: Life-limiting condition
OSCE: Objective Structured Clinical Examination
PGY: Postgraduate year
PICU: Pediatric Intensive Care Unit
R: Statistical software
SP: Standardized patient
T1: Pre-intervention time point
T2: Post-intervention time point
UTHealth: University of Texas Health Science Center at Houston
